# Progression of subclinical choroidal neovascularization in age-related macular degeneration

**DOI:** 10.1371/journal.pone.0217805

**Published:** 2019-06-04

**Authors:** Michael J. Heiferman, Amani A. Fawzi

**Affiliations:** Department of Ophthalmology, Northwestern University Feinberg School of Medicine, Chicago, Illinois, United States of America; Massachusetts Eye & Ear Infirmary, Harvard Medical School, UNITED STATES

## Abstract

**Purpose:**

To use optical coherence tomography angiography (OCTA) to study longitudinal subclinical choroidal neovascularization (CNV) changes and their correlation with progression to exudation in age-related macular degeneration (AMD).

**Methods:**

This study included a total of 34 patients with unilateral neovascular AMD who were evaluated prospectively using OCTA to detect subclinical CNV in their fellow eye. Eyes with baseline subclinical CNV were followed with serial OCTA for a minimum of one year (15.2±3.27 months) to monitor the development of exudation.

**Results:**

Of the 34 fellow eyes studied, five were found to have baseline subclinical CNV. One of the five cases of baseline subclinical CNV converted to exudative AMD during the follow up period. The average surface area of baseline subclinical CNV on OCTA was 0.131±0.096 mm^2^ which progressed to 0.136±0.104 mm^2^ at the final follow up (*P* = 0.539). Geographic atrophy grew at a rate of 0.82±1.20mm^2^/year in four eyes without subclinical CNV and 0.02mm^2^/year in one eye with subclinical CNV.

**Conclusion and importance:**

The rate of conversion to exudative AMD in eyes with subclinical CNV of 20% in our study is similar to previous reports and suggests the importance of vigilance in these eyes. The lower growth rate of geographic atrophy may suggest a protective effect of subclinical CNV that deserves further study.

## Introduction

Age-related macular degeneration (AMD) is a leading cause of irreversible vision loss in developed countries.[1] Early and intermediate AMD are defined by the presence of drusen; eyes with this feature can be further stratified by the size and number of drusen to determine their risk of progression to late AMD.[2] Late AMD is defined by the presence of choroidal neovascularization or geographic atrophy involving the center of the macula. While most patients with AMD have early or intermediate AMD, severe vision loss is most often related to late AMD.[3] Despite this potential morbidity, the mechanism for progression to late AMD remains unknown.

Histopathologic specimens of eyes with clinically diagnosed dry AMD have shown newly-formed blood vessels invading into the subretinal space.[4, 5] These authors proposed the presence of subclinical choroidal neovascularization in a subset of eyes with a clinical diagnosis of dry AMD. Furthermore, they speculated that these new blood vessels are precursors of late AMD. Studies using indocyanine-green angiography (ICG) further supported this hypothesis by demonstrating the presence of focal hyperfluorescence and plaques on ICG in fellow eyes of unilateral exudative AMD.[6] These authors suggested that eyes with these ICG findings are at higher risk of developing late AMD.

While subclinical CNV appears to be a precursor and risk factor for exudative AMD, there is some evidence to suggest a protective effect against progression of geographic atrophy. Capuano et al. evaluated cases of subclinical CNV in eyes with geographic atrophy finding a lower mean atrophy rate compared to the reported rates in all eyes with geographic atrophy.[7] This group warned against treating subclinical CNV with anti-VEGF until conversion to exudative AMD. However, the relationship between subclinical CNV and geographic atrophy needs to be explored further to determine if a subset of patients with subclinical CNV warrant treatment.

The development of optical coherence tomography angiography (OCTA) has allowed identification of subclinical CNV in eyes with dry AMD with similar effectiveness compared to ICG.[8, 9] The prevalence of subclinical CNV in fellow eyes of patients with unilateral exudative AMD has been reported to be between 6.25–27%, with our group finding a prevalence 14.7%.[8, 10–13] Given this notable prevalence of subclinical CNV, the pathophysiology of conversion to exudative AMD has important clinical implications. Our goal in the present study was to explore the longitudinal changes of subclinical CNV and geographic atrophy in fellow eyes of patients with unilateral CNV. We also wanted to determine the rate of conversion to exudative AMD in our subclinical CNV cohort.

## Materials and methods

### Study design

This was a prospective study of patients with exudative AMD seen at a single institution between October 2016 and September 2018. Patients were included in the study if one eye was diagnosed with exudative AMD and one with dry AMD, irrespective of AMD stage. Eyes with a diagnosis of exudative AMD had current subretinal or intraretinal fluid or a history of fluid requiring anti-vascular endothelial growth factor (anti-VEGF) therapy. Exclusion criteria were a prior history of anti-VEGF therapy, disciform scar in the dry AMD eye, poor image quality with signal strength below 50, or motion artifact. The study protocol was approved by the Institutional Review Board of Northwestern University and this research followed the tenets of the Declaration of Helsinki. Written informed consent was obtained from all participants.

### Image acquisition

Patients underwent complete ophthalmologic evaluation including Heidelberg Spectralis HRA + OCT confocal scanning laser ophthalmoscope, SD-OCT (Heidelberg Engineering Inc.), and OCTA (RTVue-XR Avanti system, Optovue Inc., Fremont, CA USA) with split-spectrum amplitude-decorrelation angiography (SSADA) software. Two consecutive B-scans (M-B frames), each containing 304 A-scans were captured at each sampling location and SSADA was used to extract OCTA information in a 3 x 3-mm^2^ area. The device scan rate was 70,000 scans per second with a light source of 840 nm and a full-width at half maximum bandwidth of 45nm.

### Image analysis

**Fig 1:**
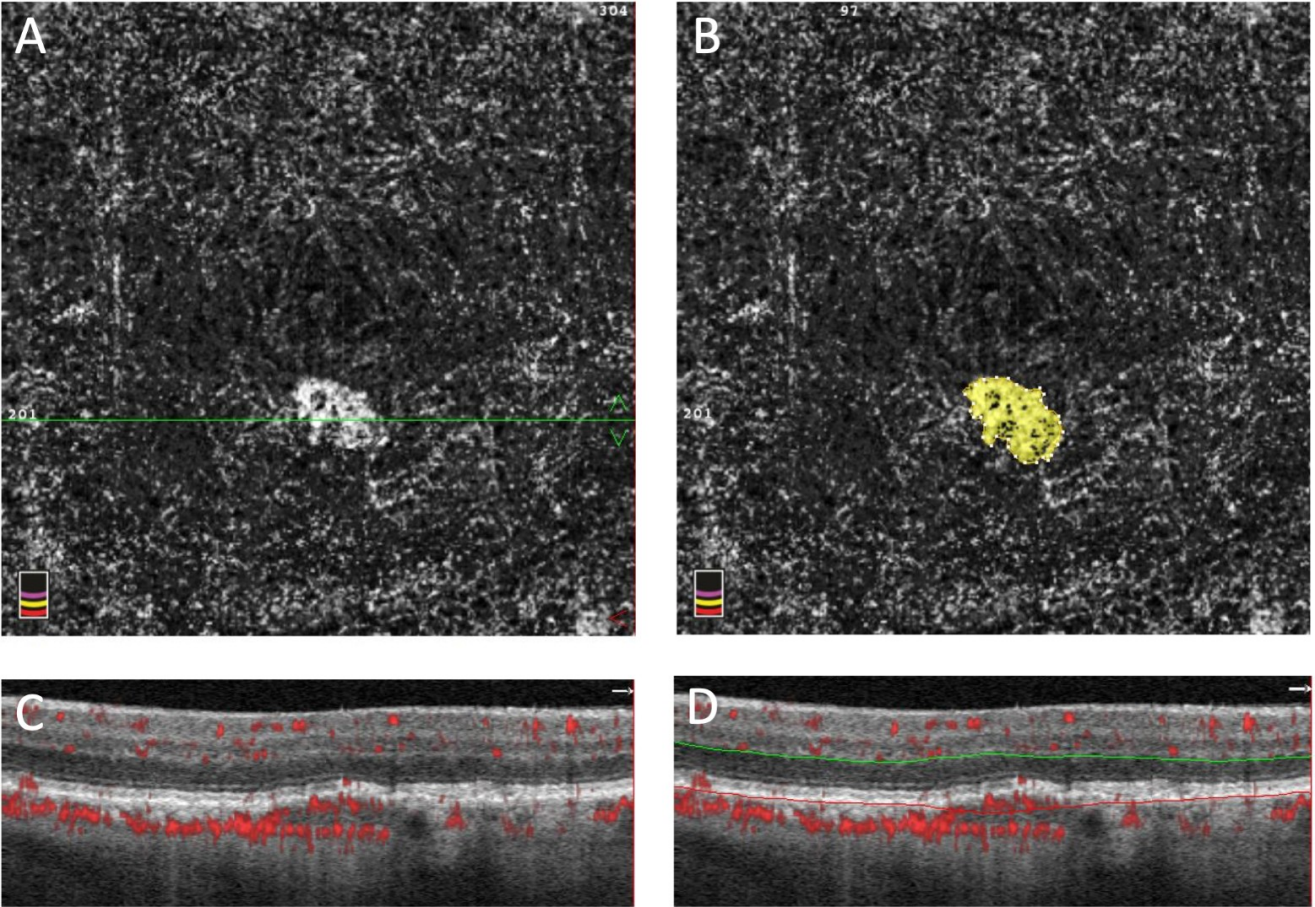
Example of subclinical CNV analysis. A: En face OCTA slab of subclinical CNV. B: Flow area selection tool used in the AngioVue software. C: OCTA B-scan with flow marked in red. The horizontal green line seen in panel A marks the section of OCTA B-scan image. D: The same OCTA B-scan segmented 72 microns below (green line) the inner plexiform layer to Bruch’s membrane (red line).

Determination of the presence of subclinical CNV was previously described by our group.[13] Briefly, an en face slab was created using AngioVue (versions 2016.1.0.26 and 2017.1.0.151) that was segmented 72 microns below the inner plexiform layer (IPL) to Bruch’s membrane (Fig 1D). Subclinical CNV was verified using the corresponding B-scans showing flow signal above Bruch’s membrane. The subclinical CNV surface area was measured using ImageJ software (NIH, Bethesda, MD) with the polygon selection tool. The flow area selection tool included in the AngioVue software was used to measure the flow area (Fig 1). The presence and location of subclinical CNV was verified using the en face slabs as well as the corresponding B-scans. The percent area choriocapillaris nonperfusion (PCAN) was calculated as previously described by our group.[14] This method uses the foveal avascular zone as a threshold to determine the areas of nonperfusion in the choriocapillaris. Whole PCAN was calculated for the entire slab while halo PCAN was calculated for the area within 200 micrometers outside of the CNV border. Conversion to exudative AMD was determined by initiation of anti-VEGF therapy and confirmed by review of fundus photos, OCT, and fluorescein angiography, when available.

Geographic atrophy (GA) was defined as an area of sharply demarcated depigmentation measuring greater than or equal to 250μm in the longest linear dimension. Detection of GA was performed by a single investigator (MJH) evaluating all fundus autofluorescence (FAF) and SD-OCT images from each patient visit. Growth of GA was measured on FAF using the selection function of ImageJ software (NIH, Bethesda, MD). The scale was determined for each image setting the distance between the center of the fovea and the center of the optic disc to 4.5mm.

## Results

**Fig 2:**
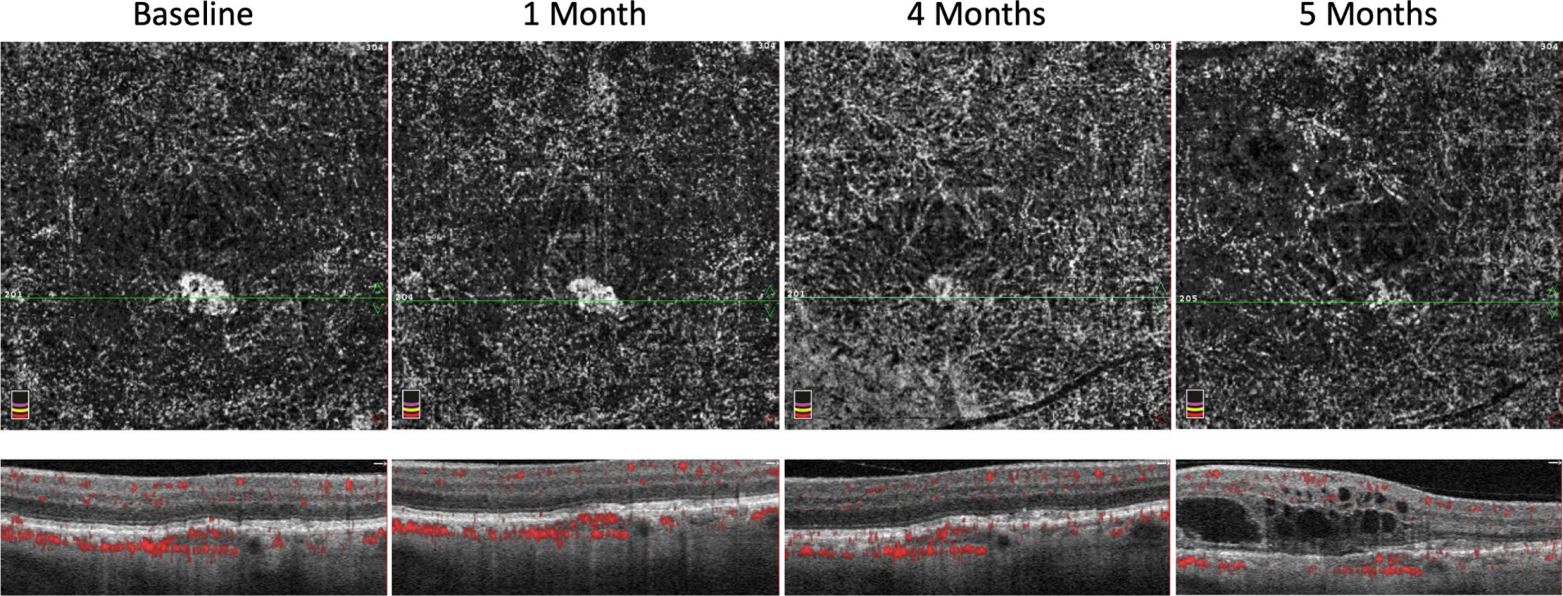
Case of subclinical CNV that converted to exudative AMD.

**Fig 3:**
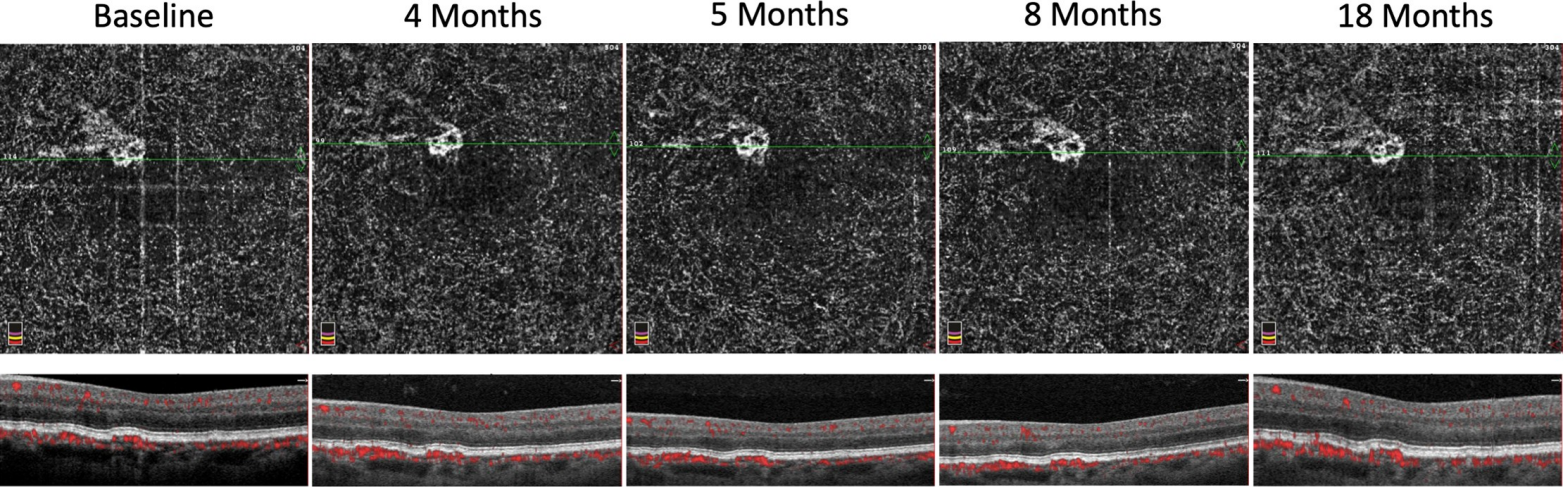
Case of subclinical CNV that remained relatively stable in surface area and flow area.

Thirty-four previously identified patients were included in this study, including five fellow eyes previously found to have subclinical CNV (14.7%). Subclinical CNV patients were followed for an average time period of 15.2±3.27 months. Patients without subclinical CNV were followed for an average time period of 12.4±5.47 months. Of the five eyes with subclinical CNV, one developed exudative AMD requiring anti-VEGF therapy initiation (20%, Fig 2). None of the twenty-nine eyes without subclinical CNV developed exudative AMD. The average surface area of subclinical CNV was 0.131±0.096 mm^2^ at baseline and 0.136±0.104 mm^2^ at the final follow up (*P* = 0.539, Table 1, Fig 3). The average flow area of subclinical CNV was 0.103±0.074 mm^2^ at baseline and 0.110±0.074 mm^2^ at the final follow up OCTA (*P* = 0.355). The single case of subclinical CNV that converted to exudative AMD increased in surface area from 0.139 mm^2^ at baseline to 0.164 mm^2^ at the final follow up OCTA.

**Table 1:** Changes in patient cohort at baseline and follow up.

	Baseline	Follow up
Surface area of subclinical CNV	0.131±0.096 mm^2^	0.136±0.104 mm^2^
Flow area of subclinical CNV	0.103±0.074 mm^2^	0.110±0.074 mm^2^
Geographic atrophy without subclinical CNV	1.68±2.20 mm^2^	2.84±3.27 mm^2^
Geographic atrophy with subclinical CNV	0.190 mm^2^	0.229 mm^2^

### Geographic atrophy

Of the thirty-four fellow eyes included in this study, one of the five eyes with subclinical CNV had GA and four of the twenty-nine eyes without subclinical CNV had GA at the time of initial imaging. The eye with subclinical CNV had a growth rate of GA of 0.02mm^2^/year compared to the average growth rate of GA in eyes without subclinical CNV of 0.82±1.20mm^2^/year. The single case of subclinical CNV that converted to exudative AMD developed GA after initiation of anti-VEGF therapy and had a growth rate of 0.95mm^2^/year. None of the remaining eyes developed new GA.

### Percent area choriocapillaris nonperfusion (PCAN)

The average whole PCAN of the subclinical CNV cases that did not convert to exudative AMD was found to be 8.43±3.03. The average halo PCAN of these four subclinical CNV cases was 10.72±3.875. These cases were not significantly different from the case of subclinical CNV that converted to exudative AMD with a whole PCAN of 11.21 and halo PCAN of 11.26. There was no correlation between conversion to exudative AMD and the initial whole PCAN or halo PCAN.

## Discussion

In the present study, longitudinal monitoring of subclinical CNV revealed a rate of progression to exudative AMD of 20% in our cohort. The rate of conversion of subclinical CNV to exudative AMD has been studied by multiple groups (Table 2). de Oliveira Dias et al. identified 23 eyes with subclinical CNV and found progression to type 1 CNV in five eyes over twelve-months (21.7%).[10] Capuano et al. followed 19 eyes with subclinical CNV and found progression to exudative AMD that required anti-VEGF therapy in five eyes over a six-month period (26%).[7] Carnevali et al. identified 15 eyes with subclinical CNV and found progression to exudative AMD in one eye over a twelve-month period (6.6%). Yanagi et al. followed 8 eyes with subclinical CNV and 10 with polypoidal choroidal vasculopathy and estimated an annual incidence of exudation of 18.1%.[15] We observed a conversion rate of 20%, consistent with the majority of reported rates, and higher compared to Carnevali et al. who observed a rate of 6.6% over one year.[9] The differences may be explained by study design, since Carnevali et al. defined quiescent CNV as treatment-naïve CNV that remained non-exudative during 6-months of follow-up. In their cohort, eyes that converted to exudative AMD prior to 6-month follow-up were excluded from further analysis, which explains their lower rate of conversion. Interestingly, the study by Capuano et al. which had the highest rate of conversion (26% over six months) only evaluated subclinical CNV in eyes with geographic atrophy, which may have led to overestimation of the rate of conversion, given their focus on eyes with late AMD.

**Table 2:** Studies evaluating rate of exudation in eyes with subclinical CNV.

Study	Eyes with Subclinical CNV	Eyes Progressed to Exudative AMD	Follow up period
Dias et al. (2017)	23	5 (21.7%)	12 months
Capuano et al. (2017)	19 (GA only)	5 (26.3%)	6 months
Carnevali et al. (2018)	15	1 (6.67%)	12 months
Yanagi et al. (2018)	18 (includes PCV)	4 (estimated 18.1% annually)	>6 months
Our study	5	1 (20.0%)	12 months

de Oliveira Dias et al. evaluated the rate of fellow eye exudation in 112 patients with unilateral exudative AMD without evidence of subclinical CNV in their fellow eye at baseline.[10] Three of these eyes progressed to exudative AMD without intervening development of subclinical CNV (2.7%), while six eyes developed subclinical CNV by one year follow up (5.4%). Upon retrospective review of their data, they identified elevations in the RPE in the three eyes without subclinical CNV that predated their progression to exudative CNV. These authors speculated that RPE elevations were precursors to exudation and that more frequent OCTA imaging may be useful to detect subclinical CNV in these eyes.

Other groups have studied longitudinal changes in eyes with subclinical CNV. Compared to baseline, Carnevali et al. observed a statistically significant increase in CNV area at 6- and 12-months follow up (1.511±1.628 mm^2^, 1.769±1.867 mm^2^, 1.930±1.964 mm^2^, respectively). While the majority of their 15 cases showed an increase in CNV area, the case that converted to exudative CNV almost doubled in size (0.284 mm^2^ to 0.541 mm^2^) at 6-months. However, when looking at their entire cohort, they did not find a difference in BCVA, central macular thickness, retinal thickness in the affected sectors, or subclinical CNV vessel density. In contrast, we did not find a significant change in CNV area in the present study, which may be explained by their larger sample size of 15 eyes. Carnevali et al. used a similar method to measure area of CNV using ImageJ software with the polygon selection tool. Additionally, we saw no change from baseline to final follow-up BCVA, surface area of CNV, flow area of CNV, whole PCAN, or halo PCAN in our cohort.

Our group previously used quantitative OCTA techniques to measure the choroidal vasculature in patients with reticular pseudodrusen finding an absence of the choriocapillaris layer, which correlated with vision in these eyes.[16] Choriocapillaris loss has been correlated with drusen density in histopathologic studies of early AMD.[17–19] However, the clinical implication of choriocapillaris loss in AMD has been a focus of intense ongoing investigation. We previously showed a greater halo PCAN in eyes with exudative AMD compared to their fellow subclinical CNV eyes.[13] However, in the current study we found no correlation between baseline area of whole or halo PCAN and development of exudative AMD in eyes with subclinical CNV. This finding suggests that choriocapillaris dysfunction may play a role in exudative AMD, but it may not be the only factor predisposing eyes to converting from subclinical to exudative CNV. Carnevali et al. described a doubling in CNV area and change in morphology that preceded exudation in their case of subclinical CNV progressing to exudation.[9] Our case of subclinical CNV that converted to exudative AMD had an increase in CNV area (0.139 mm^2^ to 0.164 mm^2^), but not the doubling nor an appreciable change in morphology. While risk factors for the development of exudative AMD in the fellow eye include large drusen, pigmentary abnormalities, and RPE elevation; none have been identified in eyes with subclinical CNV.[20, 21] Future studies with a larger series and higher resolution imaging may evaluate choriocapillaris nonperfusion adjacent to subclinical CNV as a risk factor for exudation.

Our study has several limitations, most notably, the small sample size. We were also limited by our follow up schedule, based on anti-VEGF injections in the fellow eye. This variability may have precluded our ability to detect morphologic changes in our case of subclinical CNV converting to exudative AMD.

We observed one case of subclinical CNV with associated geographic atrophy at baseline imaging. The rate of atrophy progression in this case was 0.02 mm^2^/year. This is lower than the rate of 0.82±1.20mm^2^/year in the four other cases that had baseline GA without subclinical CNV in our series. This rate is also lower than 2.79 mm^2^/year described by Sunness et al. in a large cohort of 123 eyes with geographic atrophy (their magnification adjustment factor was disc area = 2.54 mm^2^).[22] Capuano et al. evaluated cases of subclinical CNV in eyes with geographic atrophy finding a mean atrophy growth rate of 1.38±0.93 mm^2^/year.[7] They proposed that subclinical CNV had a protective effect against progression of atrophy, which was supported by their finding that the subclinical CNV bordered the area of atrophy, similar to our case. While the pathophysiology of AMD remains unknown, complement-mediated injury is thought to be promoted by heterogeneous stressors including oxidative stress.[23, 24] The potential protective effect of subclinical CNV in GA progression may be related to a relative reduction in oxidative stress. Our case of subclinical CNV converting to exudative AMD developed new geographic atrophy that grew at a rate of 0.95 mm^2^/year. This rate is similar to the other four cases of geographic atrophy without associated subclinical CNV (0.82±1.20mm^2^/year) and lower than the reported rate of all cases of geographic atrophy (1.85 mm^2^/year). However, no definitive conclusions can be drawn from our data, given our small sample size.

In the present study, we add to the body of research describing the natural history of subclinical CNV with only a subset of cases converting to exudation, without any morphometric predictive factors for conversion. Future studies evaluating the relationship between subclinical CNV and GA would aide in our understanding and management of these patients. Larger series of subclinical CNV that converted to exudative AMD may identify features that portend higher risk of exudation, which would warrant preventative treatment.

## Supporting information

S1 Dataset Spreadsheet describing the surface area and flow area of subclinical CNV cases and surface area of geographic atrophy cases. (XLSX)
